# Association between household air pollution and child mortality in Myanmar using a multilevel mixed-effects Poisson regression with robust variance

**DOI:** 10.1038/s41598-021-92193-0

**Published:** 2021-06-21

**Authors:** Juwel Rana, Rakibul M. Islam, Md Nuruzzaman Khan, Razia Aliani, Youssef Oulhote

**Affiliations:** 1 Department of Public Health, School of Health and Life Sciences, North South University, Dhaka, 1229 Bangladesh; 2 Department of Biostatistics and Epidemiology, School of Public Health and Health Sciences, University of Massachusetts, Amherst, MA 01003 USA; 3 South Asian Institute for Social Transformation (SAIST), Dhaka, 1205 Bangladesh; 4 Department of Epidemiology and Preventive Medicine, Monash University, Melbourne, VIC 3004 Australia; 5 Department of Population Sciences, Jatiya Kabi Kazi Nazrul Islam University, Mymensingh, Bangladesh; 6 School of Medicine and Public Health, Faculty of Health and Medicine, University of Newcastle, New South Wales, Australia; 7 Ministry of National Health Services, Regulation and Coordination, Islamabad, Pakistan; 8 Department of Environmental Health, T. H. Chan School of Public Health, Harvard University, Boston, MA USA

**Keywords:** Risk factors, Environmental impact

## Abstract

Household air pollution (HAP) from solid fuel use (SFU) for cooking is a major public health threat for women and children in low and middle-income countries. This study investigated the associations between HAP and neonatal, infant, and under-five child mortality in Myanmar. The study consisted of 3249 sample of under-five children in the households from the first Myanmar Demographic and Health Survey 2016. Fuel types and levels of exposure to SFU (no, moderate and high) were proxies for HAP. We estimated covariate-adjusted relative risks (aRR) of neonatal, infant, and under-five child mortality with 95% confidence intervals, accounting for the survey design. The prevalence of SFU was 79.0%. The neonatal, infant, and under-five child mortality rates were 26, 45, and 49 per 1000 live births, respectively. The risks of infant (aRR 2.02; 95% CI 1.01–4.05; p-value = 0.048) and under-five mortality (aRR 2.16; 95% CI 1.07–4.36; p-value = 0.031), but not neonatal mortality, were higher among children from households with SFU compared to children from households using clean fuel. Likewise, children highly exposed to HAP had higher risks of mortality than unexposed children. HAP increases the risks of infant and under-five child mortality in Myanmar, which could be reduced by increasing access to clean cookstoves and fuels.

## Introduction

Under-five child mortality accounts for 70 percent of the global deaths among children and young under 25 years old in 2019^1^. Of these, 2.4 million die in the first month of life and 1.5 million in the first year of life^1^. The burden of neonatal, infant, and under-five child mortality is disproportionate across regions. For instance, South-East Asian countries, including Bangladesh, Bhutan, India, Nepal, Sri Lanka, and Maldives, share one of the highest rates of under-five child mortality globally despite their progress in reducing child mortality and meeting the Millennium Development Goals (MDGs)^1,2^. However, Myanmar was unable to meet the MDGs (goal 4) to reduce child mortality. In 2019, the estimated overall under-five, infant and neonatal mortality rates were 32, 26, and 20 per 1000 live births, respectively, in South-East Asia, while Myanmar has one of the highest child mortality rates in the region, which is more than the overall rates^1,3^. Multiple underlying factors such as socioeconomic inequalities, poor sanitation and lack of safe drinking water, and poor access to clean fuels might be responsible for these high under-five and infant mortality^1–4^.

Household air pollution (HAP) from solid fuels use (SFU) is one of the world's major environmental threats, causing about 1.6–3.1 million premature deaths annually^5^. HAP related mortality is disproportionately higher in low and middle-income countries (LMICs). In 2017, almost 70% of all deaths related to HAP occurred in LMICs^6^. About 3 billion people use solid fuels for cooking, including coal and biomass (wood, animal dung, lignite, charcoal, straw/shrubs, grass, and agricultural crop)^7,8^, which are the major sources of HAP^9^.

Alternative fuels (clean fuels) such as liquefied petroleum gas and electricity are often unavailable and/or unaffordable in LMICs^10^. Therefore, households opt to collect solid fuels^7^, which are burned indoors in conventional cookstoves as a pit, pieces of brick, or U-shaped mud construction. Duflo et al. illustrate via energy ladder that households with the lowest income levels use the most inefficient and the most polluting types of fuel^11^. These solid fuels emit damaging airborne pollutants, including Particulate Matter (PM), NOx, CO, SOx, formaldehyde, and many toxic polycyclic aromatic hydrocarbons and other organic matter due to inefficient combustion^12–14^. The amount of exposure to an individual in such settings has been measured to be much higher than the World Health Organization (WHO) guidelines and standards^15^.

In LMICs, women and children are at higher risk of exposure to HAP^16–18^ due to women's role in household chores, cooking, and caring for infants in most South-East Asian cultures. Women spend about three to seven hours per day near the stove, sometimes carrying their infants for care and warmth during cooking, leading to children being exposed to biomass fuel at similar levels^7^. This exposure level increases in households with limited ventilation and poor design of the stove that do not have flues or hood to move out the smoke from living places^19^.

The majority of households in Myanmar use solid fuels for cooking, as there is easy access to biomass fuels^20^. The Clean Cooking Alliance estimated that more than 95% of the rural and 88% of the urban population use solid fuels for cooking in Myanmar^20^, which might be one of the contributing factors of more than 3500 annual infant and child deaths from acute lower respiratory infections (ALRIs) and pneumonia in Myanmar. It could also be one of the reasons that prevented Myanmar from achieving the MDGs (between 2000 and 2015) of reducing infant and child mortality^2,4^. Importantly, this indicates an important area of intervention for achieving the Sustainable Development Goals (SDGs) of reducing neonatal (12 per 10,000 live births) and infant (25 per 10,000 live births) deaths between 2015 and 2030.

To our knowledge, no study evaluated the effect of HAP from SFU on neonatal, infant, and under-five mortality rates in Myanmar using nationally representative data. The first Demographic Health Survey (MDHS) in Myanmar was conducted in 2016 and provided an opportunity to examine the associations of HAP with neonatal, infant, and under-five child mortality.

## Methods

### Study design and setting

Given the focus on improving maternal and child health, the MDHS 2016 was the first nationally representative cross-sectional survey conducted in Myanmar. Data were collected from 12,885 women from the sampled households based on stratified two-stage cluster sampling design from December 2015 to July 2016. Using the 2014 Myanmar census sampling units, 442 clusters (123 urban, 319 rural) were selected in the first stage from 4000 clusters based on the probability proportional to the size. In the second stage, 30 households from each selected cluster were selected in the first stage by using systematic random sampling. The overall response rate was approximately 98%. The survey was funded by the United States Agency for International Development and implemented by the Ministry of Health and Sports, Myanmar, in coordination with the MDGs. Technical support was provided by ICF international. Details of the survey sampling procedure have been published in the MDHS report^21^.

### Characteristics of participants

**Figure 1:**
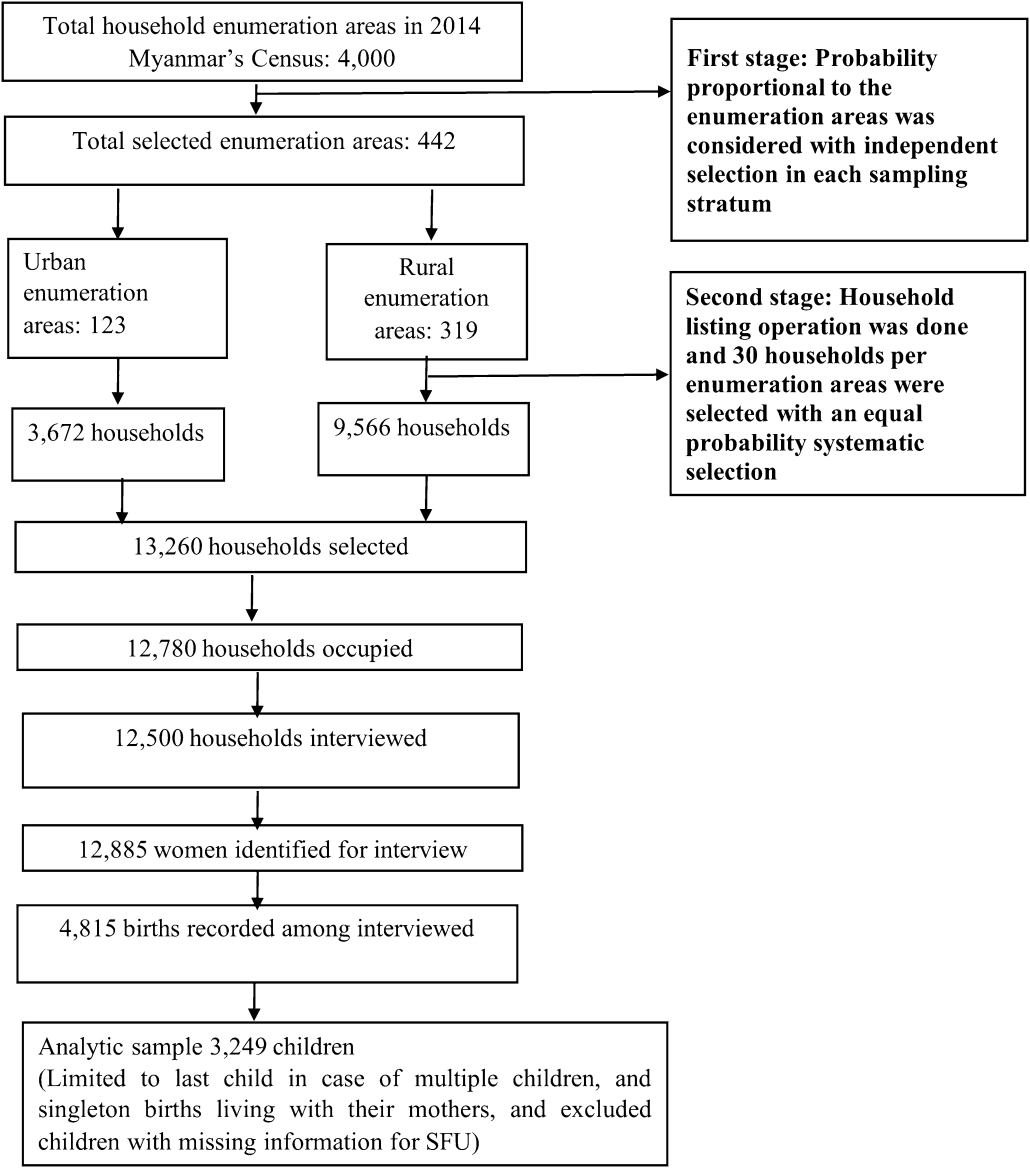
Schematic of the analytic sample selection process for child mortality in Myanmar.

A total of 3249 under-five children were included in the final analysis based on their retrospective birth histories after limiting to singleton births living with their mothers at the time of the survey and excluding children with missing information on SFU (Fig. 1)^21,22^. The inclusion criteria were: (i) children born within five years before the date of survey (only last child and singleton births were considered in case of multiple children in five years); (ii) most recent children with information of survival status (alive/death at the time of the survey); (iii) children with the date of death if applicable; (iv) children with complete information of household cooking fuels use^21^.

### Measures of child mortality outcomes

We considered neonatal mortality (deaths occurred during the first 28 days of life), infant mortality (deaths occurred during the first one year (0–11 months) of life), and under-five mortality (deaths occurred during the first five years (0–59 months) of life) as outcome variables^21,23,24^.

### Measures of HAP exposure

The analysis was carried out for two exposure indicators: SFU (clean fuel *vs.* solid fuel) and levels of exposure to SFU induced HAP (no exposure, moderate exposure, and high exposure). The MDHS collected information on the types of cooking fuels by asking women—*what type of fuel does your household mainly use for cooking?* Responses were coded as clean fuel = 0 (if responses were electricity, liquid petroleum gas, and natural gas) and solid fuel = 1 (if responses were coal, lignite, charcoal, wood, straw/shrubs, grass, agricultural crop, and others). Children's levels of exposure to HAP were generated from the women's responses to the place of cooking and the type of cooking fuel use^23–26^. The responses were categorized as no exposure = 0 (if women reported not using solid fuel), moderate exposure = 1 (if women reported using solid fuel, but in a separate building or outdoors), and high exposure = 2 (if women reported using solid fuel inside the house).

### Confounder selection and adjustment

**Figure 2:**
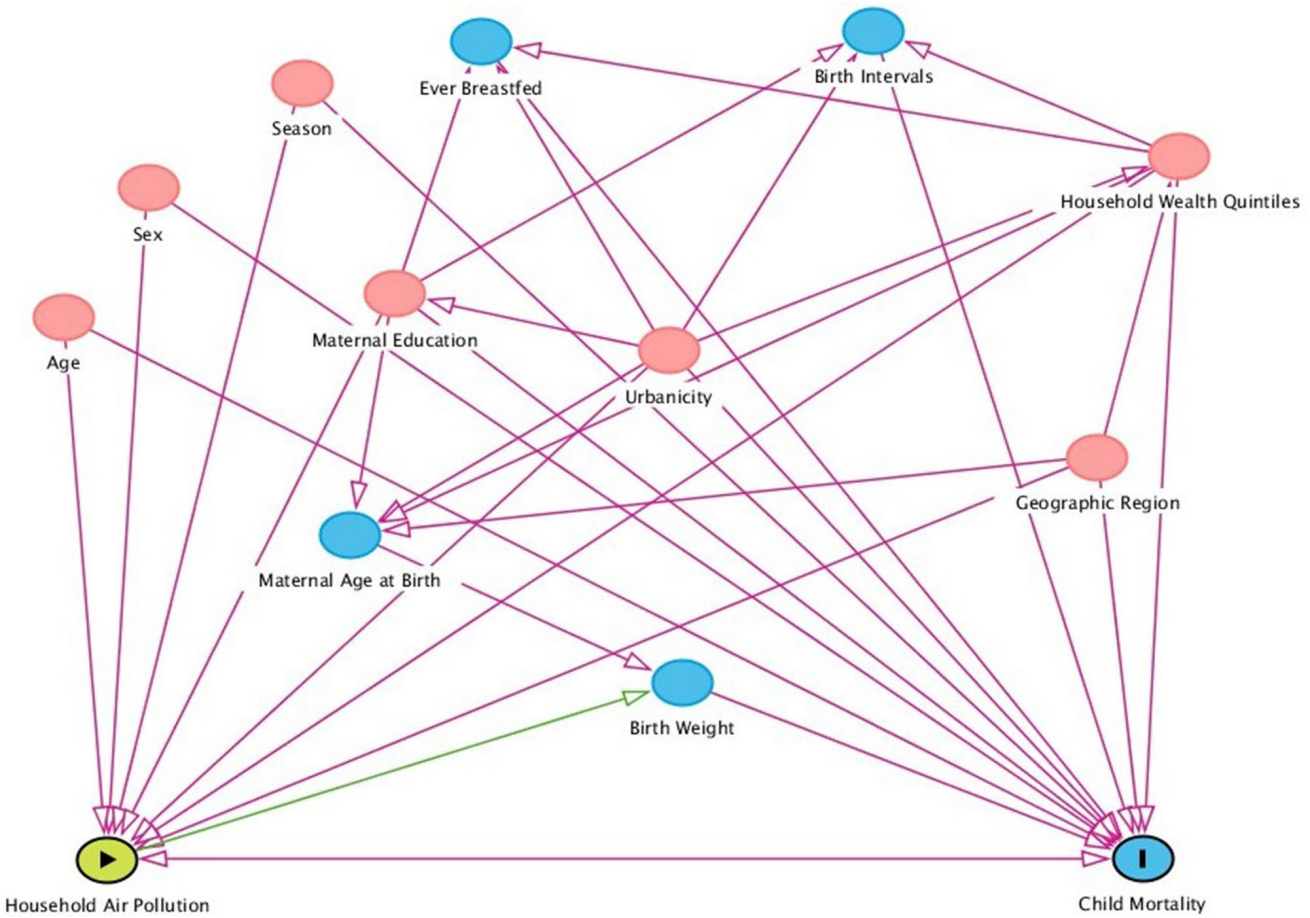
A directed acyclic graph (DAG) for evaluation of covariates selection in the analysis of effects of HAP on child mortality. HAP is exposure, and child mortality is the outcome. The minimal and sufficient adjustment set contains child age, child sex, breastfeeding status, maternal education, household wealth quintiles, urbanicity, geographic region, preceding birth interval, and season. This figure was constructed through DAGitty (http://www.dagitty.net).

Different sociodemographic factors contributing to the neonatal, infant, and under-five child mortality were included as confounders (Fig. 2). These were age at child deaths, child sex, parental education, interval of last two succeeding births, breastfeeding status, household wealth quintiles, urbanicity, geographic regions, and seasons (Fig. 2). The birth interval variable was generated based on women's response to the birth date of the last two children and categorized by following the WHO guidelines^21^. The wealth quintile was reconstructed from the women's household durable and non-durable assets (e.g., televisions, bicycles, sources of drinking water, sanitation facilities, and construction materials of houses) using principal components analysis, excluding the types of cooking fuels to avoid over adjustment as this was the main exposure of interest^21,26^.

### Statistical analysis

Descriptive statistics were reported as frequency and percentage to characterize the demographic profile of the study sample. Differences in neonatal, infant, and under-five child mortality across sociodemographic factors were presented using the chi-square test. The associations between exposure to HAP and child mortality outcomes were investigated using both univariable and multilevel mixed-effects Poisson regression models. As an additional analysis, effect modification by sex of the child was also tested in all models by including a cross-product term between child sex and HAP. The univariate models included only the exposure variable and the outcome variable. These associations were then adjusted for potential confounders in the multivariable models, including child age, child sex, breastfeeding status, maternal education, household wealth quintiles, urbanicity, geographic region, preceding birth interval, and season. However, birth weight was not included in the models as it is likely to be on the causal pathway between exposure to HAP and mortality^27–29^. Furthermore, information on exact birth weight was unavailable for most of the children^21^.

Multilevel mixed-effects Poisson regression models with robust error variance were used to avoid overestimation of associations with common binary outcomes measured in cross-sectional study^22,26,30^. We also accounted for complex survey design effects^22,26^. Results were reported as relative risks (RRs) with 95% confidence intervals (CIs). All statistical analyses were two-sided, and a *p*-value < 0·05 was considered statistically significant. The ICF Institutional Review Board (IRB) and the Ministry of Health and Sports, Myanmar, approved the primary data collection survey protocol. Informed consent was taken from each participant before the survey. We obtained the de-identified data and public-use dataset from the DHS online archive. We followed Strengthening the Reporting of Observational Studies in Epidemiology (STROBE) guidelines to design and report the results^31^. All methods were performed in accordance with the relevant guidelines and regulations.

## Results

Characteristics of the participants, exposures, and outcomes are presented in Table 1. The mean (±SD) age of the mothers was 31.1 (± 6.0) years. The mean years of education were 4.4 (± 3.5) years. The mean age of the child was 2.1 (± 1.4) years, and 47.6% of children were girls. More than three-quarters (78.8%) of the study households used solid fuels (charcoal 16.1%, wood 60.5%, agricultural crop 1.2%, and others 1%) for cooking, of which 62.3% used solid fuels at indoor cooking places. About two-thirds (65.1%) of the women reported indoor place of cooking. Nearly half of the children (47.7%) were highly exposed to HAP during the survey (Table 1).

**Table 1:** Key information about the study participants, exposure, and outcome variables.

Demographics of mothers	Frequency (n = 3249)	Weighted percentage (95% CI)
Mean age in years (mean ± SD)	3249	31.1 (± 6.0)
Mean weight in kilograms (mean ± SD)	3249	53.9 (± 10.9)
Mean years of education (mean ± SD)	3249	4.4 (± 3.5)
**Demographics of under-five children**		
Mean age in years (mean ± SD)	3249	2.1 (± 1.4)
Girls	1559	47.6 (45.4–49.8)
**Types of cooking fuels**		
Electricity	675	20.8 (18.2–23.7)
Liquid petroleum gas + natural gas	14	0.4 (0.2–0.8)
Charcoal	522	16.1 (14.0–18.4)
Wood	1966	60.5 (56.9–64.0)
Agricultural crop	40	1.2 (0.8–2.0)
Coal, lignite + straw/shrubs/grass + others	32	1.0 (0.7–1.4)
**Cooking place**		
Indoor	2099	65.1 (62.0–68.1)
Separate building	763	23.7 (21.0–26.5)
Outdoors	362	11.2 (9.9–12.7)
**Exposure to household air pollution**		
Solid fuel use	2560	78.8 (75.8–81.5)
Clean fuel use	689	21.2 (18.5–24.2)
Indoor solid fuel use	1579	62.3 (58.7–65.7)
**Levels of exposure to household air pollution**		
Unexposed	689	22.4 (19.1–26.1)
Moderate exposure	956	29.9 (26.8–33.0)
High exposure	1579	47.7 (43.9–51.6)
**Outcomes**		
Neonatal mortality per 1000 live births	89	26.0 (19.0–35.0)
Infant mortality per 1000 live births	144	45.0 (35.0–57.0)
Under-five mortality per 1000 live births	158	49.0 (38.0–62.0)

*SD* Standard deviation, *CI* confidence interval.

The rate of neonatal, infant, and under-five child mortality was 26 (95% CI 19–53), 45 (95% CI 35–57), and 49 (95% CI 38–62) per 1000 live births, respectively (Table 1). Infant and under-five child mortality were slightly higher in girls, while neonatal mortality was higher in boys. A similar increasing trend was observed for infant and under-five child mortality for rural residents. Compared with ever (not current) breastfeeding status, neonatal (415.6, 95% CI 279.5–565.9), infant (465.8, 95% CI 325.3–611.9) and under-five child mortality (465.8, 95% CI 325.3–611.9) per 1000 live births were higher amongst mothers who never breastfeed. Infant and under-five child mortality were higher among children whose mothers had no education, resided in Shan, Chin, and Teninthayi regions, and were born in the short birth interval (Table 2).

**Table 2:** Neonatal, infant, and under-five child mortality rates by sociodemographic and spatial factors (weighted).

Sociodemographic and spatial factors	Neonatal mortality per 1000 (95% CI)	Infant Mortality per 1000 (95% CI)	Under-five mortality per 1000 (95% CI)
**Maternal age at birth**			
≤ 24 years	24 (13.4–42.9)	44.1 (28.0–68.7)	48.1 (31.4–72.9)
25–35 years	25.4 (18.1–35.5)	41.9 (32.1–54.6)	44.0 (33.9–57.0)
More than 35 years	34.2 (22.6–51.5)	50.1 (35.4–70.4)	59.8 (43.7–81.4)
**Sex of the children**			
Male	27.0 (19–39.1)	44.0 (32.1–60.1)	48.2 (35.1–65.2)
Female	25.1 (16.2–40.1)	46.4 (33.3–63.1)	49.2 (36.2–67.3)
**Breastfeeding status**			
Never	415.6 (279.5–565.9)	465.8 (325.3–611.9)	465.8 (325.3–611.9)
Ever	26.9 (18.7–38.6)	59.2 (44.2–78.7)	66.0 (49.2–88.1)
**Maternal education**			
None	44.2 (28.3–68.4)	77.4 (52.2–112.4)	83.3 (55.1–124.3)
Primary	18.4 (11.2–31.4)	36.1 (25.2–52.2)	40.0 (29.1–56.3)
Secondary	21.4 (12.0–37.3)	29.3 (18.1–48.3)	30.0 (19.0–49.1)
Higher	51.3 (15.2–161.3)	51.2 (15.2–161.1)	51.2 (15.1–161.1)
**Household wealth quintiles**			
Poorest	19.3 (08.1–39.2)	40.4 (24.0–66.3)	42.3 (26.0–68.1)
Poor	21.4 (11.0–41.4)	32.4 (19.4–55.5)	38.2 (22.1–66.3)
Middle	32.0 (18.3–56.4)	48.2 (31.6–73.4)	54.2 (36.1–80.1)
Richer	27.3 (16.1–48.2)	55.3 (35.1–85.2)	59.2 (39.2–90.1)
Richest	28.3 (15.3–49.4)	45.2 (29.2–71.2)	45.2 (29.1–71.6)
**Urbanicity**			
Urban	28.3 (16.6–48.4)	43.8 (27.5–68.6)	46.4 (29.3–71.5)
Rural	26.4 (18.5–36.6)	46.6 (34.0–61.6)	50.3 (37.2–66.4)
**Geographic region**			
Kachin	24.4 (10.5–57.4)	03.1 (01.4–07.0)	35.5 (16.5–75.3)
Kayah	22.5 (11.4–45.7)	02.9 (01.5–05.6)	29.5 (15.4–57.4)
Kayin	19.6 (05.0–71.9)	31.0 (12.7–76.4)	35.4 (14.3–86.5)
Chin	53.4 (36.5–79.5)	75.3 (53.0–106.4)	83.3 (55.4–122.1)
Sagaing	24.3 (08.5–69.3)	28.5 (11.3–72.4)	32.3 (13.3–76.6)
Tenintha	17.5 (06.5–50.4)	52.3 (20.4–127.9)	69.6 (32.2–143.2)
Bago	21.4 (08.0–57.4)	33.2 (15.1–69.6)	33.2 (15.3–69.4)
Magway	24.4 (10.5–59.6)	37.6 (19.8–67.4)	43.2 (22.4–84.6)
Mandalay	13.5 (03.5–50.4)	38.4 (16.4–86.4)	38.8 (16.6–86.4)
Mon	18.3 (07.3–45.3)	37.1 (16.4–81.4)	43.5 (18.1–101.1)
Rakhine	33.5 (14.6–76.6)	38.6 (18.6–76.4)	38.4 (18.3–76.4)
Yangon	27.3 (06.1–119.4)	43.1 (14.5–122.5)	43.4 (14.2–122.5)
Shan	38.2 (20.0–70.6)	79.5 (45.5–135.5)	84.5 (45.6–151.4)
Ayeyarwa	32.1 (13.0–73.5)	55.6 (28.4–103.6)	60.0 (32.4–108.5)
Naypyiataw	07.5 (0.9–40.4)	20.0 (06.0–56.5)	20.4 (07.4–57.4)
**Birth interval**			
First birth	20.7 (12.9–33.2)	31.9 (21.8–46.3)	35.0 (24.6–49.7)
≥ 24 months	23.1 (16.4–33.3)	39.3 (30.4–50.5)	43.4 (33.6–55.5)
< 24 months	47.5 (25.4–84.5)	83.5 (51.8–131.1)	88.6 (55.4–136.6)
**Seasons**			
Summer (March–April)	15.6 (08.4–28.5)	46.4 (28.5–75.0)	51.5 (30.5–87.4)
Rainy (May–July)	10.4 (03.5–40.6)	18.7 (0.7–40.6)	20.6 (08.3–47.5)
Winter (December–February)	33.3 (23.4–46.3)	48.6 (36.4–64.6)	51.5 (39.4–67.5)

The unadjusted and adjusted associations between HAP and child mortality are presented in Table 3 (Supplementary Fig. 1). The risks of infant mortality (2.02, aRR 95% CI 1.01–4.05; p-value = 0.048) and under-five mortality (aRR 2.16, 95% CI 1.07–4.36; p-value = 0.031) were two times higher in children from households who used solid fuel for cooking compared to children from households who used clean fuel. The risks were even higher when we considered the augmented measure of exposure to HAP. Compared with unexposed children, infant mortality risks were 1.94 (95% CI 0.92–4.08; p-value = 0.081) and 2.15 (95% CI 1.04–4.43; p-value = 0.038) times higher among moderately and highly HAP exposed children, respectively.

**Table 3:** Associations between HAP exposure and risk of neonatal, infant, and under-five child mortality in Myanmar.

Exposures	Neonatal mortality	*p*-value	Infant mortality	*p*-value	Under-five mortality	*p*-value
	RR (95% CI)		RR (95% CI)		RR (95% CI)	
**Unadjusted**						
**Exposure to household air pollution**						
Clean fuel	1.00		1.00		1.00	
Solid fuel	1.53 (0.69–3.38)	0.298	1.59 (0.85–2.99)	0.147	1.77 (0.94–3.32)	0.078
**Levels of exposure to household air pollution**						
Unexposed	1.00		1.00		1.00	
Moderate	1.72 (0.73–4.08)	0.219	1.66 (0.82–3.33)	0.158	1.83 (0.93–3.61)	0.080
High	1.41 (0.63–3.15)	0.406	1.56 (0.83–2.94)	0.169	1.73 (0.91–3.31)	0.094
**Adjusted**^a^						
**Exposure to household air pollution**						
Clean fuel	1.00		1.00		1.00	
Solid fuel	0.95 (0.64–1.40)	0.780	2.02 (1.01–4.05)	0.048	2.16 (1.07–4.36)	0.031
**Levels of exposure to household air pollution**						
Unexposed	1.00		1.00		1.00	
Moderate	0.96 (0.66–1.39)	0.829	1.94 (0.92–4.08)	0.081	2.11 (1.02–4.40)	0.045
High	1.02 (0.67–1.54)	0.938	2.15 (1.04–4.43)	0.038	2.25 (1.08–4.69)	0.030

*RR* relative risks, *CI* confidence interval.

^a^Multilevel Mixed-effects Poisson Regression models were adjusted for child age, child sex, breastfeeding status, maternal education, household wealth quintiles, urbanicity, geographic region, preceding birth interval and season.

A similar higher risks of under-five mortality was observed among children with moderate (aRR 2.11; 95% CI 1.02–4.40; p-value = 0.045) and high (aRR 2.25, 95% CI 1.08–4.69; p-value = 0.030) exposure to HAP than their counterparts. There was no association between neonatal mortality with HAP exposure and levels of exposure to HAP. As an additional analysis (not shown), we did not observe effect modification by child sex in the associations between exposure to HAP and levels of exposure and mortality outcomes of under-five children.

## Discussion

The first-ever nationally representative survey suggests that neonatal, infant, and under-five child mortality rates were relatively higher in Myanmar compared with other South-east Asian countries^2,3,23^. Most of the households were dependent on SFU for cooking, and almost half of the study children were highly exposed to HAP in Myanmar. The study demonstrates that HAP and moderate and high levels of exposure to HAP increased the risk of infant and under-five child mortality, but not neonatal mortality in Myanmar.

Previous studies reported comparable results that HAP exposure from SFU increases the risk of infant and child mortality in LMICs^22,23,32–34^. Evidence suggests that the combustion of SFU emits multiple pollutants such as fine particles, carbon monoxide, formaldehyde, and many more toxic chemicals, which increase the risk of mortality from ALRIs, asthma, and pneumonia among infants and young children exposed to these pollutants^7,12,13,26,35–40^. Exposure to these toxic pollutants also increases the risk of stillbirth, low birth weight, and preterm birth, including acute and chronic health problems, all of which are considered leading causes of child mortality^3,22,23,41^.

Previous studies suggest considering cooking place along with SFU to examine its effects on child mortality because cooking inside the house with solid fuels maximizes the concentrations of airborne toxic pollutants in the household and ambient air^23–26^. We employed an augmented SFU exposure measure combining SFU and cooking place following the previous study and found stronger effects of high exposure to HAP on infant and child mortality^26^. The high prevalence of SFU suggests that children in this study were exposed to high concentration of pollutants as found in other studies, which suggest that high proximity to pollutants and spending much time in the kitchen during heating and cooking intensify the risk of adverse health outcomes, including child mortality from ALRI^26,32,35^. The plausible explanation is that young children are more susceptible to HAP-induced mortality than their older counterparts due to their underdeveloped epithelial linings of the lungs^26,42^. Furthermore, infants at their early age are often carried on their mothers' backs or placed to sleep or stand beside their mother when cooking, a common practice in South-east Asian countries, including Myanmar ^23,24,43,44^.

In a healthy condition, infants and young children have higher respiration rates, and they breathe 50% more polluted air due to their narrower airways and large lung surface. Children have a weak immune system in their early years of life; thus, HAP exposure might increase the risk of child mortality from ALRI through impaired airway and systemic immunity, airway inflammation, etc.^35,42,45,46^.

However, neonatal mortality was not significantly associated with SFU and exposure to HAP in our study, consistent with previous studies conducted in LMICs^32,47^. Several biological factors, such as low birth weight, prematurity, and complications associated with pregnancy and delivery, might be responsible for the null association between HAP and neonatal mortality^3,23,41^. Maybe the effects of these risk factors are much stronger that it became more difficult to disentangle the effects of HAP on neonatal mortality. However, it needs further investigation using longitudinal studies with measures of air pollutants. Additionally, breastfeeding could work as a protective factor diminishing the effect of HAP on neonatal mortality. Moreover, neonates and mothers might live in a conducive environment right after delivery, as well as mothers usually stay away from any cooking activities during the neonatal period, which is a common cultural practice in Asia. However, few studies claim that neonates are at higher risk of HAP induced mortality^22,44^, which warrant further studies.

The main strength of the study was a nationally representative survey with a 98% response rate. The analysis of large-scale data with an appropriate statistical method and adjustments for potential confounders makes the study findings valid for policymaking. However, the main weakness is that the temporal association between HAP exposure and child mortality outcomes cannot be established due to its cross-sectional nature. Second, the associations could be affected by unmeasured confounders and different health outcomes such as preterm birth, low birth weight, and other morbidity factors despite HAP exposure. Third, information related to the children's birth and death was reported by mothers that may introduce recall biases and errors. However, it is unlikely that the mother would incorrectly report their children's birth and death, although there could be errors in the time of death that would likely lead to non-differential misclassification. Fourth, exposure measurement error is very likely as we used two proxy measures such as SFU and combining SFU and cooking place to measure the associations between HAP exposures^26^ and child mortality. However, this is the available robust and established measurement of HAP exposures because DHS does not objectively measure the level and duration of HAP exposures^24,26^. Further studies may include questions related to ventilation in the kitchen, duration of cooking, proximity to the kitchen, or heating areas to better measure children's exposure to HAP.

## Conclusion

The study suggests that HAP is a significant risk factor for infant and under-five child mortality but not neonatal mortality. Furthermore, both moderate and high levels of exposure to HAP, such as the combination of SFU and cooking inside the kitchen, increase infant and child mortality risk in Myanmar. The results from this study should be corroborated by longitudinal studies with objective measures of air pollutants. If confirmed, policymakers should take both short-term and long-term strategies through socio-environmental pathways to address the higher rate of child mortality in Myanmar, which will ultimately help them meet several SDGs.

## Supplementary Information

Supplementary Figure 1.

## Data Availability

Myanmar Demographic Household Survey (MDHS) data were obtained from the MEASURES DHS. The datasets generated and/or analyzed during the current study are available in the 2015–16. https://dhsprogram.com/pubs/pdf/FR324/FR324.pdf.

## Abbreviations

HAP: Household air pollution
SFU: Solid fuel use
LMICs: Low and middle-income countries
MDHS: Myanmar demographic and health survey
WHO: World Health Organization
ALRIs: Acute lower respiratory infections
MDGs: Millennium development goals
SDGs: Sustainable development goals
DHS: Demographic health survey

## Code availability

All analyses were carried out using statistical software packages Stata version 16.0^48^.
