# Supplementary figures and images for: Association between household air pollution and child mortality in Myanmar using a multilevel mixed-effects Poisson regression with robust variance
Source: Sci Rep. 2021 Jun 21;11:12983. doi: 10.1038/s41598-021-92193-0 (PMC8217172; doi:10.1038/s41598-021-92193-0)

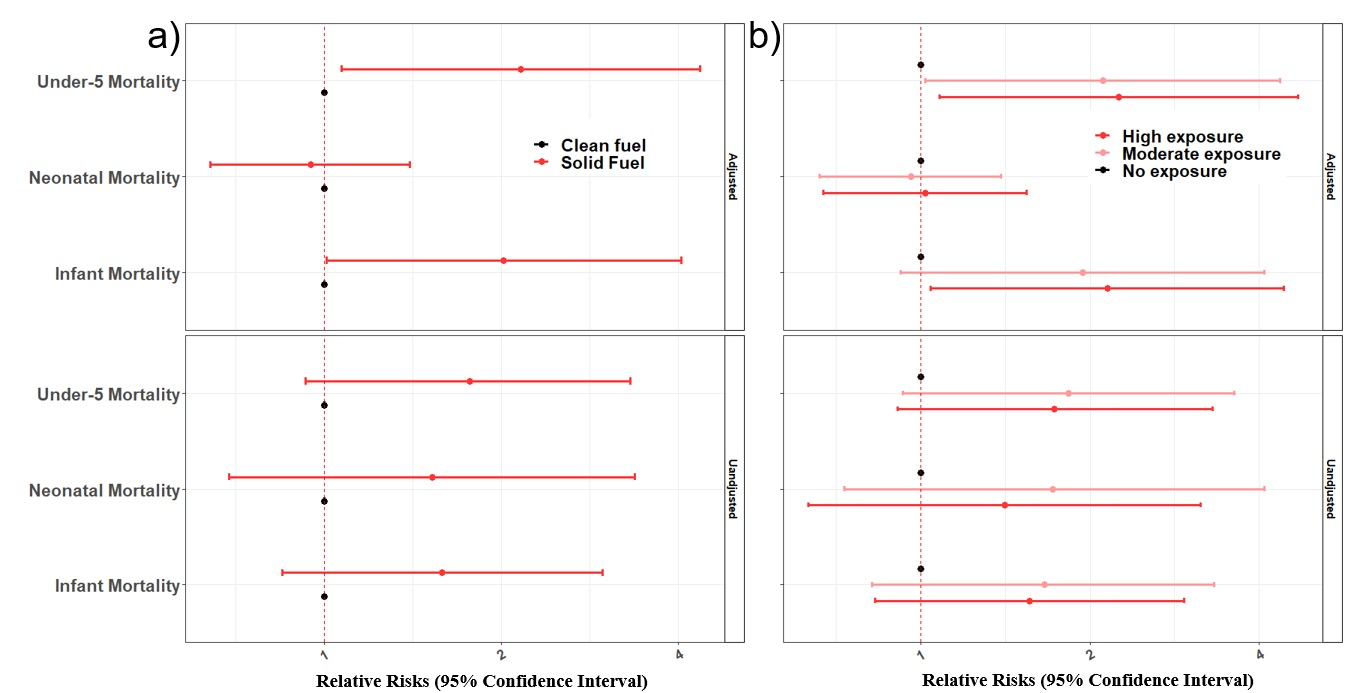

Supplement: Supplementary file 1 — Supplementary Figure 1. [file 41598_2021_92193_MOESM1_ESM.png]
